# Distinct Pattern of Atypical Megakaryocytes in VEXAS Syndrome

**DOI:** 10.1111/ijlh.70097

**Published:** 2026-03-26

**Authors:** Andrew Y. Sung, Jackson W. Douglas, Olga K. Weinberg, Sharon K. Germans, Weina Chen

**Affiliations:** ^1^ Department of Pathology University of Texas Southwestern Medical Center Dallas Texas USA

VEXAS (vacuoles, E1 enzyme, X‐linked, autoinflammatory, somatic) syndrome is an autoinflammatory syndrome caused by somatic pathogenic mutations in the X‐linked *UBA1* (ubiquitin‐like modifier activating enzyme 1) gene. This entity typically presents in the seventh decade of life and with a strong male predominance. In addition to rheumatologic manifestations, patients often have hematologic abnormalities such as macrocytic anemia, thrombocytopenia, and/or monocytopenia. Key morphologic findings in the bone marrow (BM) include vacuoles in the myeloid and erythroid precursors, often in a hypercellular BM with granulocytic hyperplasia and mild morphological dysplasia [1, 2, 3]. Karyotypes are often normal while gene alterations, largely associated with age‐related clonal hematopoiesis, are present in a small subset of patients (e.g., *TET2* [8%] and *DNMT3A* [15%]) [2]. Progression to myelodysplastic syndrome/neoplasm (MDS) is common, with up to 50% of patients also carrying a diagnosis of MDS [1]. Here, we present a case of VEXAS syndrome with distinctly atypical megakaryocytes and gene alterations that blurred the diagnostic line between VEXAS syndrome and MDS.

The patient was a 59‐year‐old male who initially presented with macrocytic anemia (hemoglobin: 6.5 g/dL, MCV: 119 fL), thrombocytopenia (platelet count [plt]: 94 × 10^9^/L), headache, eye pain, and an elevated erythrocyte sedimentation rate. Together, the constellation of cytopenia and autoinflammatory symptoms should raise suspicion for VEXAS syndrome or an evolving MDS. *UBA1* mutation analysis of the peripheral blood (PB) sample detected a *UBA1* p.Met41Thr (p.M41T) mutation (82% variant allele frequency [VAF]) disrupting normal translation of the cytoplasmic UBA1b isoform and leading to defective protein degradation [3]. Following initial diagnosis of VEXAS syndrome, the patient was treated with azacitidine and tocilizumab. PB examination at 1 year after initial presentation showed mild macrocytic anemia (hemoglobin: 11.2 g/dL, MCV: 118 fL), thrombocytopenia (Plt: 96 × 10^9^/L), and normal neutrophil and monocyte counts (2.45 and 0.30 × 10^9^/L, respectively). The BM was hypercellular (95% cellularity) with granulocytic hyperplasia (M:E ratio: 4.7) and left‐shifted erythroid maturation, vacuolated myeloid and erythroid precursors, and distinctly atypical megakaryocytes, including condensed eosinophilic globules in the cytoplasm, and peripherally displaced, “wreath‐like” nuclei (Figure 1). No significant dysplasia in other myeloid and erythroid lineage cells or increased blasts (2% by manual differential) was observed. Iron studies on the BM aspirate showed normal storage iron and no ring sideroblasts. Flow cytometry of the BM specimen did not reveal any immunophenotypic aberrancies. In the BM specimen, cytogenetic studies showed a normal karyotype (46,XY) and a targeted next‐generation sequencing panel revealed likely pathogenic mutations in *TET2* (p.Ser152fsTer1, 50% VAF and p.Glu1555fsTer23, 7% VAF), *TLL2* (p.Arg986Ter, 51% VAF), and *ZRSR2* (p.Gln32fsTer6, 12% VAF).

**FIGURE 1:**
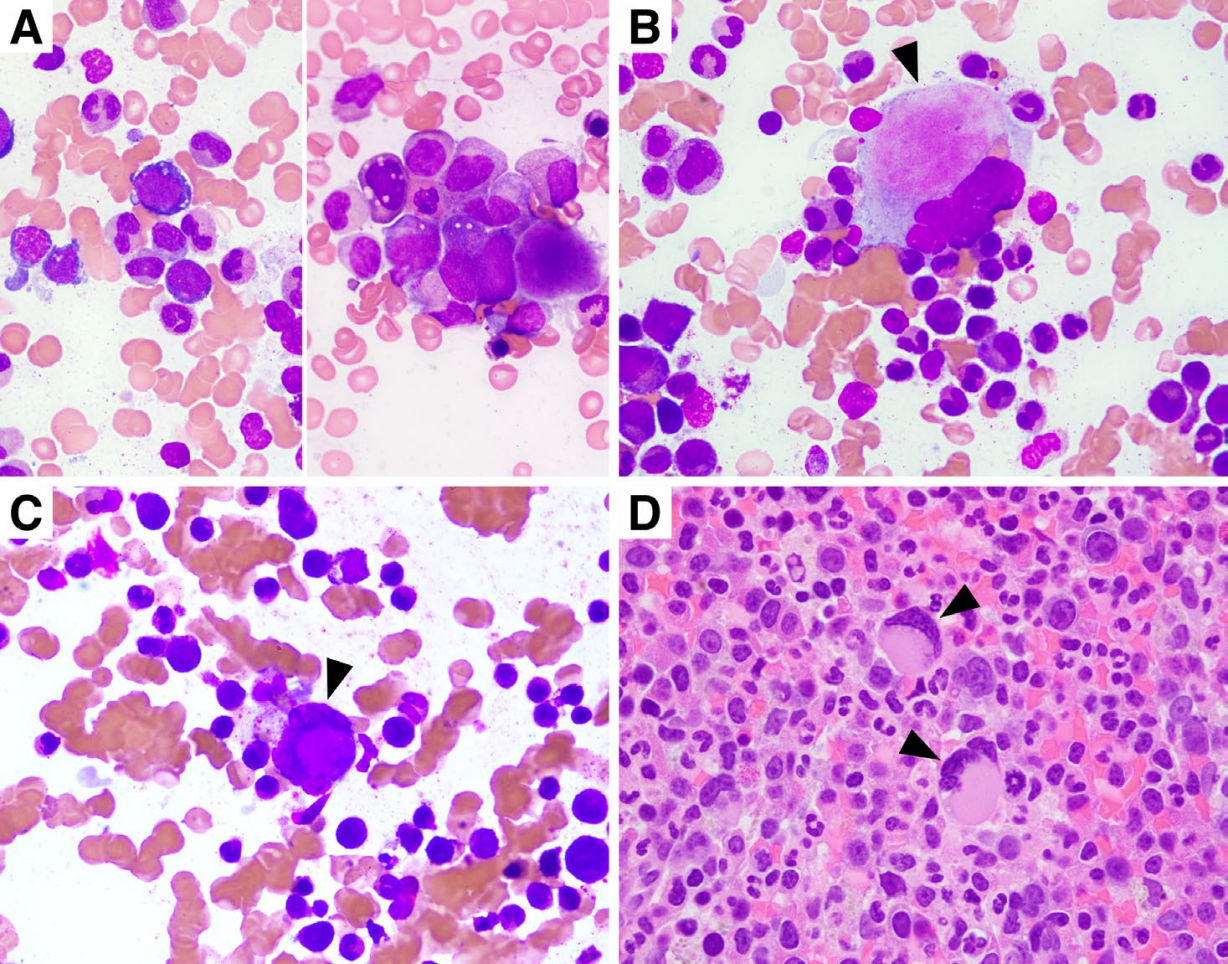
Distinct pattern of atypical megakaryocytes in VEXAS syndrome. Bone marrow (BM) aspirate (A; Wright‐Giemsa‐stain, 1000×) showing vacuolization in erythroid precursors (left‐A) and myeloid precursors (right‐A). BM aspirate (B and C; Wright‐Giemsa‐stain, 1000×) and core biopsy (D; hematoxylin–eosin stain, 500×) showing megakaryocytes with condensed eosinophilic globule in the cytoplasm (arrowheads, B) and peripherally displaced, “wreath‐like” nuclei (arrowheads, C and D).

Given the underlying dysplastic features in VEXAS syndrome, the diagnosis of MDS should be approached with caution. Indeed, some patients with VEXAS syndrome have been misclassified as MDS [2]. In this patient, aside from vacuolization in the myeloid and erythroid precursors, dysplasia was limited to the megakaryocytes with the distinct pattern of atypia recently described in VEXAS syndrome (i.e., condensed eosinophilic globules in the cytoplasm and peripherally displaced, “wreath‐like” nuclei) [2]. In conjunction with the *UBA1* p.Met41Thr mutation, low blast count, no significant dysplasia in other myeloid and erythroid lineages, unremarkable flow cytometry findings, and absence of MDS‐defining mutations or cytogenetic abnormalities, we considered the spectrum of findings to be most consistent with VEXAS syndrome. While the gene alterations in *TET2* and *ZRSR2* may be found in MDS, they are not MDS‐defining (e.g., *TP53* and *SF3B1*) and have also been reported in VEXAS syndrome [2]. However, the presence of these co‐mutations may increase the risk of progression to MDS in this patient.

Ultimately, this case illustrates the importance of recognizing the distinct pattern of atypical megakaryocytes with vacuolated myeloid and erythroid precursors in the setting of cytopenia and autoinflammation in order to prompt genetic testing for *UBA1* mutation and avoid misdiagnosing VEXAS syndrome as MDS.

## Author Contributions

J.W.D., O.K.W., S.K.G., and W.C. performed the diagnostic workup for this case. W.C. provided the images of the bone marrow aspirate and core biopsy. A.Y.S. and W.C. wrote the manuscript.

## Funding

The authors have nothing to report.

## Ethics Statement

This article does not contain any studies with human participants or animals performed by any of the authors.

## Consent

Informed consent has been obtained from the patient.

## Conflicts of Interest

The authors declare no conflicts of interest.

## Data Availability

Data sharing is not applicable to this article as no datasets were generated or analyzed during the current study.
